# Diagnostic efficacy of ultrasound and computed tomography for acute appendicitis: A single center retrospective study

**DOI:** 10.1097/MD.0000000000041968

**Published:** 2025-03-28

**Authors:** Yiying Zhao, Hanwen Liu, Dechao Guo

**Affiliations:** a Special Inspection Department, The Third Affiliated Hospital of Zhejiang, Chinese Medical University, Hangzhou, China; b Department of General Surgery, The Third Affiliated Hospital of Zhejiang Chinese Medical University, Hangzhou, Zhejiang Province, China.

**Keywords:** acute appendicitis, CT, diagnosis, negative appendectomy, ultrasound

## Abstract

The aim of this study was to evaluate the diagnostic efficacy of ultrasonography (US) and computed tomography (CT) examination for acute appendicitis (AA). A total of 41 patients with suspected AA were enrolled in this study. CT scan was performed in 24 patients, and US was performed in 17 patients. Both CT scan and US were performed in 7 patients. The primary outcomes were the performance characteristics (sensitivity, specificity) of US and CT in the patients with suspected AA. The secondary outcomes included the diagnostic accuracy of CT and US. By using US, 6 patients (35%) were incorrectly diagnosed and 2 other patients (12%) had equivocal results. By using CT as the primary diagnostic tool, 4 patients (17%) were misdiagnosed and 8 patients (33%) had equivocal results. Even if the equivocal results of CT and US were excluded from the calculation, the sensitivity and specificity of CT was 88% and 71% respectively, while the sensitivity and specificity of US was 73% and 50%, respectively. Although CT and US are believed to be reliable diagnostic tool to precisely diagnose AA. The rate of negative appendectomy still remains high. There is a need to develop a more accurate methods to diagnose AA, and therefore rate of negative appendectomy can be reduced.

## 1. Introduction

Acute appendicitis (AA) is one of the most common surgical emergencies, yet its preoperative diagnosis remains challenging. Despite advancements in medical imaging, the rate of negative appendectomies remains high due to the continued reliance on clinical presentation for diagnosis.^[1,2]^ Although ultrasonography (US) and computed tomography (CT) are widely used diagnostic tools, recent studies suggest that their implementation has not significantly reduced false diagnoses of AA.^[3–5]^ Misdiagnosis may lead to delayed management or unnecessary appendectomy, both of which pose risks to patient safety and healthcare resource utilization.^[6,7]^ Thus, improving diagnostic accuracy is crucial for optimizing patient outcomes.

A significant gap in the literature exists regarding the comparative effectiveness of US and CT when used together for diagnosing AA. While most studies have evaluated the diagnostic accuracy of each modality independently, few have directly assessed their combined diagnostic value in a clinical setting.^[8,9]^ Additionally, some researchers have proposed colonoscopy as a potential diagnostic tool for AA, but concerns regarding its safety and the limited availability of supporting evidence have prevented its widespread adoption.^[10,11]^ The lack of comprehensive comparative studies creates uncertainty about the most effective diagnostic approach to minimize unnecessary surgical interventions.

This study aims to evaluate the diagnostic efficacy of US and CT in the preoperative diagnosis of AA. By assessing their sensitivity and specificity both individually and in combination, this study seeks to provide evidence-based insights that could refine diagnostic strategies and reduce false diagnoses. The findings may contribute to optimizing imaging modalities in clinical practice, ultimately enhancing diagnostic accuracy and improving patient care.^[12,13]^

## 2. Material and methods

### 2.1. Patients and study protocols

The consecutive patients with suspected AA were enrolled in this study between December 2021 and December 2023. The inclusion criteria included: patients clinically suspected with AA (Alvarado scores ≥ 5)^[14]^; patients with a AA diagnosed by abdominal CT or US; and for patients such as pregnant women, children, and couples planning for conception who rejected CT, the diagnosis was confirmed by colonoscopy. Exclusion criteria included those with perforated appendicitis or periappendiceal abscess confirmed by non-enhanced CT (16-detector-row) or US.

The study was performed according to the principles of the Declaration of Helsinki, and was approved by the Institutional Review Board of Binhai County People’s Hospital.

### 2.2. Outcomes and definitions

The primary outcomes were the performance characteristics (sensitivity, specificity) of US and CT in the patients with suspected AA. The secondary outcomes included the diagnostic accuracy of CT and US.

False positive diagnosis of AA was defined, if patients were diagnosed as AA on the initial examination by US or CT, however, final diagnosis was not AA when both US or CT or alternative examination was performed.^[15]^ Patients without appendicitis (NA) mean that patients were not diagnosed as AA and finally diagnosed as other disease, uneventfully recovered or relieved with a stable condition after corresponding treatment based on the guidelines.

### 2.3. Follow up

Telephone follow-up was conducted for those patients who were not diagnosed as AA and were discharged after conservative treatment of their symptoms. If any symptoms present, such as abdominal pain, fever or other digestive symptoms, the patient would be recommended for further examinations in clinic (physical examinations, laboratory tests and US or CT, if necessary).

### 2.4. Statistical analyses

Results were expressed as mean ± standard deviation (SD) or median (inter-quartile range, IQR). Quantitative variables were compared between the 2 groups using the *t t*est or the Mann–Whitney *U* test, as appropriate. Categorical variables were compared between the 2 groups using the Fisher’s exact test. A 2-tailed *P* value < .05 was considered to be statistically significant. Statistical analysis was performed using the Statistical Package for the Social Sciences (SPSS) version 17.0 for Windows.

## 3. Results

A total of 41 patients (30 males, 11 females) were included in this study. The average age of the study population was 46 ± 16.5 years. The median Alvarado score was 6 (IQR: 5–7.5) in patients with clinically suspected acute appendicitis (AA). Among them, 29 patients (70%) were definitively diagnosed with AA using ultrasound (US) or computed tomography (CT), while 12 patients (30%) were excluded from having AA. The alternative diagnoses in these 12 patients included abdominal pain of unknown etiology (n = 4), pelvic infection (n = 3), mucinous adenocarcinoma (n = 2), ileocecal lipoma (n = 2), and ulcerative colitis (n = 1). No complications were observed in any patient during the median follow-up period of 12 months (IQR: 6–24 months). The baseline characteristics of all patients are summarized in Table 1.

**Table 1:** Baseline characteristics of all the included patients.

Characteristics	Patients, n (n = 41)
Age (yr), mean ± SD	46 ± 16.5
Male/female, n	30/11
RLQ pain, n (%)	41 (100%)
RLQ tenderness, n (%)	35 (85%)
RLQ rebound pain, n (%)	25 (62%)
Body temperature (°*C*), mean ± SD	37.4 ± 0.2
WBC (*×*10^9^/L), mean ± SD	13.6 ± 0.9
CT, n	24
US, n	17
Both CT and US, n	7
Alvarado scores	8 (7–8)

CT = computed tomography, RLQ = right lower quadrant pain, US = ultrasonography, WBC = white blood cells.

In total, 24 patients underwent CT, while 17 underwent US. Both modalities were performed in 7 patients. US misdiagnosed 6 patients (35%) and yielded equivocal results in 2 patients (12%). CT misdiagnosed 4 patients (17%) and produced equivocal findings in 8 patients (33%). After excluding equivocal cases, the sensitivity and specificity of CT were 88% and 71%, respectively, whereas those of US were 73% and 50%, respectively (Table 2).

**Table 2:** Radiographics results and accuracy of CT and US.

Radiographics	Total number	Patients with acute appendicitis	Patients without appendicitis
CT scan	24	17	7
AA	11	9 (53%)	2 (29%)
NA	5	2 (12%)	3 (42%)
Equivocal	8	6 (35%)	2 (29%)
US	17	11	6
AA	11	8 (73%)	3 (50%)
NA	4	3 (27%)	1 (17%)
Equivocal	2	0	2 (33%)

AA = acute appendicitis, CT = computed tomography, NA = no appendicitis, US = ultrasonography.

## 4. Discussion

Acute appendicitis (AA) is one of the most common acute abdominal emergencies.^[16]^ However, its clinical presentation is often atypical, leading to frequent diagnostic errors. As a result, the reported rate of negative appendectomies in the literature ranges from 10% to 20%.^[17]^ In this study, we evaluated the diagnostic value of US, CT, or both in accurately diagnosing AA.

Some previous studies have suggested that colonoscopy may serve as a useful diagnostic tool for AA, particularly in patients with atypical abdominal pain or non-diagnostic imaging findings.^[18,19]^ Colonoscopic features indicative of AA include hyperemia, bulging at the appendiceal orifice, surrounding mucosal edema, and pus drainage from the appendiceal orifice. However, some patients with AA may lack these features, making colonoscopy an unreliable sole diagnostic tool. Furthermore, colonoscopy does not provide a complete assessment of the appendiceal lumen, increasing the risk of missed diagnoses. Due to these limitations and the lack of standardized diagnostic criteria, colonoscopy has not been widely adopted for diagnosing AA. Most physicians and surgeons continue to rely on CT and US as the primary imaging modalities for definitive diagnosis.^[20,21]^

The sensitivity and specificity of CT and US have been extensively evaluated in previous studies. For instance, Ullah et al reported equivocal rates of 28% for CT and 75% for US, findings that are consistent with the CT results in our study.^[22]^ However, the equivocal rate of US in our study (33%) was lower than that reported by Wilson et al, which may be attributed to the small sample size and the operator-dependent nature of US. The accuracy of US depends largely on the skill of the technician and the interpretation by the radiologist. While CT is considered more objective, its diagnostic accuracy is still limited by technical and interpretative challenges, particularly regarding the shape and position of the appendix. In fact, previous studies have reported negative appendectomy rates of up to 22% despite CT imaging.^[23]^ Another factor contributing to the relatively lower accuracy of CT and US in our study is that many cases underwent both imaging modalities due to initial equivocal results.

Many researchers advocate for the development of more reliable diagnostic methods beyond CT, given its limitations. One key concern is that CT exposes patients to ionizing radiation, which is associated with an increased risk of radiation-induced cancer.^[24]^ Additionally, CT is not a suitable imaging modality for certain patient populations, such as pregnant women or individuals planning pregnancy.^[25]^ Although US is a safer alternative, its efficacy remains low, leading to a high rate of unnecessary appendectomies.

A novel diagnostic approach, endoscopic retrograde appendicography (ERA), was introduced by Liu et al^[26]^ as a potential alternative for diagnosing AA. This technique combines colonoscopy and fluoroscopy to provide a definitive diagnosis. Their study demonstrated a remarkable 94% success rate with ERA. This method allows for direct visualization of lumen dilation, partial stenosis, and intraluminal filling defects, thereby enhancing diagnostic reliability. However, while their findings suggest that ERA could be a promising alternative to CT and US, their study lacked a direct comparison between ERA, CT, and US, highlighting the need for further research to assess their comparative diagnostic accuracy.

This study has several strengths. It provides valuable insight into the diagnostic accuracy of US and CT, comparing their effectiveness both individually and in combination. The study also contributes to the growing body of research emphasizing the need for improved imaging techniques for AA diagnosis. Furthermore, by discussing alternative diagnostic approaches such as ERA and colonoscopy, this study opens avenues for future research on nontraditional imaging methods.

However, this study has certain limitations. The sample size is relatively small, which may affect the generalizability of the findings. Additionally, the operator-dependent nature of US may introduce variability in diagnostic accuracy across different clinical settings. Another limitation is the lack of direct comparison with newer diagnostic modalities, such as MRI or AI-assisted imaging techniques, which are gaining attention in recent literature.

Future research should focus on large-scale, multi-center trials to validate these findings and determine the optimal diagnostic approach for AA. Additionally, prospective studies comparing ERA, CT, and US would provide more definitive evidence regarding their relative accuracy. Given the increasing interest in AI-based diagnostic models, future studies could also explore the integration of AI algorithms in imaging interpretation to improve diagnostic precision and reduce the rate of equivocal cases.

## Author contributions

**Conceptualization:** Hanwen Liu, Dechao Guo.

**Data curation:** Yiying Zhao.

**Formal analysis:** Yiying Zhao.

**Investigation:** Hanwen Liu.

**Methodology:** Hanwen Liu.

**Supervision:** Dechao Guo

**Validation:** Hanwen Liu.

**Writing – original draft:** Yiying Zhao, Hanwen Liu, Dechao Guo.

**Writing – review & editing:** Hanwen Liu, Dechao Guo.
